# Association of *TERT* polymorphisms with chronic hepatitis B in a Chinese Han population

**DOI:** 10.18632/oncotarget.23905

**Published:** 2018-01-03

**Authors:** Guoxia Ren, Xu Liu, Zhendong Yu, Jingjie Li, Fanglin Niu, Tianbo Jin, Jikui Liu, Mingwei Chen

**Affiliations:** ^1^ Department of Respiratory and Critical Care Medicine, The First Affiliated Hospital of School of Medicine of Xi’an Jiaotong University, Xi’an 710061, China; ^2^ Department of Intergrated Traditional Chinese and Western Medicine, Xi’an Chest Hospital, Xi’an 710100, China; ^3^ Hepato-Pancreato-Biliary Surgery, Peking University Shenzhen Hospital, Guangdong, Shenzhen 518036, China; ^4^ Central Laboratory, Peking University Shenzhen Hospital, Guangdong, Shenzhen 518036, China; ^5^ Key Laboratory of Resource Biology and Biotechnology in Western China (Northwest University), Ministry of Education, School of Life Sciences, Northwest University, Xi’an, Shaanxi 710069, China

**Keywords:** chronic hepatitis B, TERT, single nucleotide polymorphisms (SNPs), case-control, Chinese Han

## Abstract

In this study, we investigated the association between the polymorphisms of telomerase reverse transcriptase (*TERT*) gene and the risk of chronic hepatitis B (CHB) in a Chinese Han population. Four single nucleotide polymorphisms (SNPs) in *TERT* (rs10069690, rs2242652, rs2853677 and rs2853676) were genotyped from 224 CHB patients and 300 healthy controls using the Sequenom Mass-ARRAY platform. We used genetic model, haplotype analyses, chi-square test, logistic regression analysis to evaluate the association between SNPs and CHB risk. The relative risk was estimated by odd ratios (ORs) and 95% confidence intervals (CIs). We found that rs10069690 was significantly associated with an increased CHB risk in the dominant model (adjusted OR = 1.70, 95% CI: 1.06–2.71, *P* = 0.031) and additive model (adjusted OR = 1.62, 95% CI: 1.09–2.41, *P* = 0.018). The haplotype “TA” (rs10069690 and rs2242652) was found to be associated with an increased risk of CHB (adjusted OR = 1.58, 95% CI: 1.05–2.38, *P* = 0.027). Our results suggested potential genetic contributes for *TERT* in CHB development in a Chinese Han population. Future functional and association studies with larger sample sizes are required to confirm these findings.

## INTRODUCTION

Hepatitis B virus (HBV) infection is a serious public health problem worldwide, with approximately 2 billion people having a history of HBV infection and 350 million of them suffering from chronic HBV infection [1]. Based on a national epidemiological survey, it has been estimated that the weighted prevalence of hepatitis B surface antigen (HBsAg) was roughly 7.18% in Chinese population [2]. Although the host factors (such as infection age, gender and immune status), viral and environmental factors are thought to be involved in affecting the development of CHB. The mechanisms underlying the different clinical outcomes of HBV infection have not been fully understood. Studies indicated that host genetic factors play a critical role in the development of HBV infection, especially single nucleotide polymorphisms (SNPs), is regarded to be one of the determinants for this clinical heterogeneity [3, 4]. Some polymorphisms have been reported to be involved in susceptibility to CHB in disease severity and progression, or disease prognosis [4, 5].

Telomeres, located at the ends of eukaryotic chromosomes, play a crucial role in maintaining the integrity of chromosome and stability of the genome [6, 7]. Telomeres are approximately 10–15 kb in human somatic cells, and they shorten by 50–200 bp after each cycle of mitotic division [8]. In general, the incomplete replication of linear DNA molecules and gradual shortening of telomere length lead to regulated cell senescence and apoptosis or cell death. It has been reported that telomere length is associated with chronic liver diseases [9], CHB and hepatocellular carcinoma [10]. Interestingly, previous studies demonstrated that a number of polymorphisms in telomere biology gene (telomerase reverse transcriptase, *TERT*) are significantly associated with telomere length [11–13]. In addition, a recent report indicated the genotype TT of *TERT* polymorphism rs2736098 is associated with a decreased risk of CHB in Chinese males [14].

The *TERT* gene, located at human chromosome band 5p15.33, consists of 16 exons and 15 introns spanning about 35 kb. As the reverse transcriptase catalytic subunit of telomerase, the TERT is essential for the maintenance of telomere DNA length in chromosomes [15]. The activation of telomerase plays a key role in cellular immortalization and the malignant transformation of human cells. This activation requires the TERT catalyst [16]. A number of studies have also reported that variants of *TERT* gene are associated with a significantly higher susceptibility to several cancers [17–20]. However, to date, little study is reported on the association between genetic variations in the *TERT* gene and the risk of CHB. In this study, we conducted a case-control study consisting of 242 CHB patients and 300 healthy controls to investigate the association between the four common genetic variants in *TERT* and the risk CHB in a Chinese Han population.

## RESULTS

### Participants

The basic characteristic of the participants are shown in Table 1, including gender, age, smoking and drinking. A total of 242 CHB patients (188 males and 54 females) with a mean age of 50.04 (± 12.048) years and 300 healthy controls (180 males and 120 females) with a mean age of 60.42 (± 5.143) years were enrolled in the study. There were significant differences in gender, smoking, drinking and age distribution between the CHB cases and control groups (*P* < 0.05) (Table 1). In order to eliminate those residual confounding effects, the variable of gender, smoking, drinking and age were adjusted in later multivariate unconditional logistic regression analysis.

**Table 1:** Basic characteristic of the participants

Characteristic		Case (*N* = 242)	Frequency	Control (*N* = 300)	Frequency	*P*-value
Gender	female	54	22.3%	120	40.0%	< 0.001
	male	188	77.7%	180	60.0%	
Smoking	Yes	126	52.1%	89	29.7%	< 0.001
	No	116	47.9%	189	63.0%	
Drinking	Yes	90	37.2%	79	26.3%	0.033
	No	152	62.8%	199	66.3%	
Age	years (mean ± SD)	50.04 ± 12.048		60.42 ± 5.143		< 0.001

SD: Standard deviation *P* < 0.05 indicates statistical significant. The smoking and drinking information of the 22 cases in the control group were missing.

### Allele distributions

The detailed data of each SNP in *TERT* gene are shown in Table 2. Our data indicated that all four SNPs investigated were in Hardy-Weinberg equilibrium in the control subjects (*P* > 0.05). We compared the differences in frequency distributions of alleles between cases and controls by Chi-squared test. No associations were observed between the alleles of the four SNPs and CHB risk in the allele model. We also performed a Bonferroni correction and determined that none of the four SNPs showed statistical significant associations with CHB risk.

**Table 2:** Basic characteristic of the four SNPs in *TERT*

SNP-ID	Position	Band	Role	Alleles A/B	HWE	MAF		OR (95% CI)	*P*
						case	control		
rs10069690	1279790	5p15.33	Intron	T/C	0.347	0.187	0.144	1.37 (0.99–1.90)	0.054
rs2242652	1280028	5p15.33	Intron	A/G	0.523	0.181	0.160	1.16 (0.84–1.59)	0.357
rs2853677	1287194	5p15.33	Intron	G/A	0.696	0.366	0.332	1.16 (0.91–1.50)	0.234
rs2853676	1288547	5p15.33	Intron	T/C	0.817	0.158	0.147	1.09 (0.78–1.52)	0.611

SNP: Single nucleotide polymorphism, A: Minor alleles, B: Major alleles, HWE: Hardy-Weinberg equilibrium, MAF: Minor allele frequency, OR: Odds ratio, 95% CI: 95% Confidence interval.

### Genetic models

We further assessed the association between each SNP and CHB risk in an unconditional logistic regression analysis, which was performed using four models: genotype, dominant, recessive, and additive model (Table 3). The SNP rs10069690 was found to be associated with an increased risk of CHB in the dominant model (OR = 1.48, 95% CI: 1.02–2.14, *P* = 0.040; adjusted OR = 1.70, 95% CI: 1.06–2.71, *P* = 0.031) both before and after the adjustment for age, gender, smoking and drinking; and in the additive model (adjusted OR = 1.62, 95% CI: 1.09–2.41, *P* = 0.018) after the adjustment for age, gender, smoking and drinking. No significantly statistical associations were found under the other models.

**Table 3:** Genetic models analyses of the association between the SNPs and CHB risk

SNP-ID	Model	Genotype	Case	Control	OR (95% CI)	*P*	OR (95% CI)	*P*^a^
rs10069690	Codominant	TT	8	8	1.37 (0.50–3.72)	0.539	2.73 (0.84–8.88)	0.096
		TC	75	69	1.49 (1.01–2.19)	0.043	1.60 (0.98–2.60)	0.061
		CC	160	219	1.00	-	1.00	-
	Dominant	TT-TC	83	77	1.48 (1.02–2.14)	0.040	1.70 (1.06–2.71)	0.027
		CC	160	219				
	Recessive	TT	8	8	1.23 (0.45–3.32)	0.689	2.39 (0.74–7.72)	0.144
		TC-CC	235	288				
	Additive	---	---	---	1.37 (0.99–1.89)	0.057	1.62 (1.09–2.41)	0.018
rs2242652	Codominant	AA	8	9	1.16 (0.44–3.08)	0.763	2.05 (0.64–6.55)	0.225
		AG	72	78	1.21 (0.83–1.76)	0.333	1.22 (0.76–1.98)	0.415
		GG	163	213	1.00	-	1.00	-
	Dominant	AA-AG	80	87	1.20 (0.83–1.73)	0.325	1.29 (0.82–2.05)	0.274
		GG	163	213				
	Recessive	AA	8	9	1.10 (0.42–2.90)	0.846	1.94 (0.61–6.12)	0.262
		AG-GG	235	291				
	Additive	---	---	---	1.16 (0.84–1.59)	0.362	1.30 (0.88–1.93)	0.190
rs2853677	Codominant	GG	28	31	1.28 (0.72–2.28)	0.398	1.81 (0.87–3.77)	0.114
		GA	122	137	1.26 (0.88–1.81)	0.203	1.43 (0.91–2.25)	0.121
		AA	93	132	1.00	-	1.00	-
	Dominant	GG-GA	150	168	1.27 (0.90–1.79)	0.178	1.49 (0.97–2.31)	0.070
		AA	93	132				
	Recessive	GG	28	31	1.13 (0.66–1.94)	0.658	1.49 (0.75–2.98)	0.256
		GA-AA	215	269				
	Additive	---	---	---	1.18 (0.91–1.52)	0.222	1.37 (0.99–1.91)	0.058
rs2853676	Codominant	TT	3	7	0.55 (0.14–2.17)	0.395	1.42 (0.26–7.73)	0.683
		TC	71	74	1.24 (0.84–1.81)	0.277	1.23 (0.76–1.98)	0.398
		CC	170	219	1.00	-	1.00	-
	Dominant	TT-TC	74	81	1.18 (0.81–1.71)	0.393	1.24 (0.78–1.97)	0.370
		CC	170	219				
	Recessive	TT	3	7	0.52 (0.13–2.04)	0.349	1.35 (0.25–7.30)	0.728
		TC-CC	241	293				
	Additive	---	---	---	1.09 (0.78–1.53)	0.605	1.22 (0.80–1.87)	0.362

SNP: Single nucleotide polymorphism, OR: Odds ratio, 95% CI: 95% Confidence interval aP were adjusted by age, gender, smoking and drinking. *P* < 0.05 indicates statistical significant.

### Haplotype

**Figure 1:**
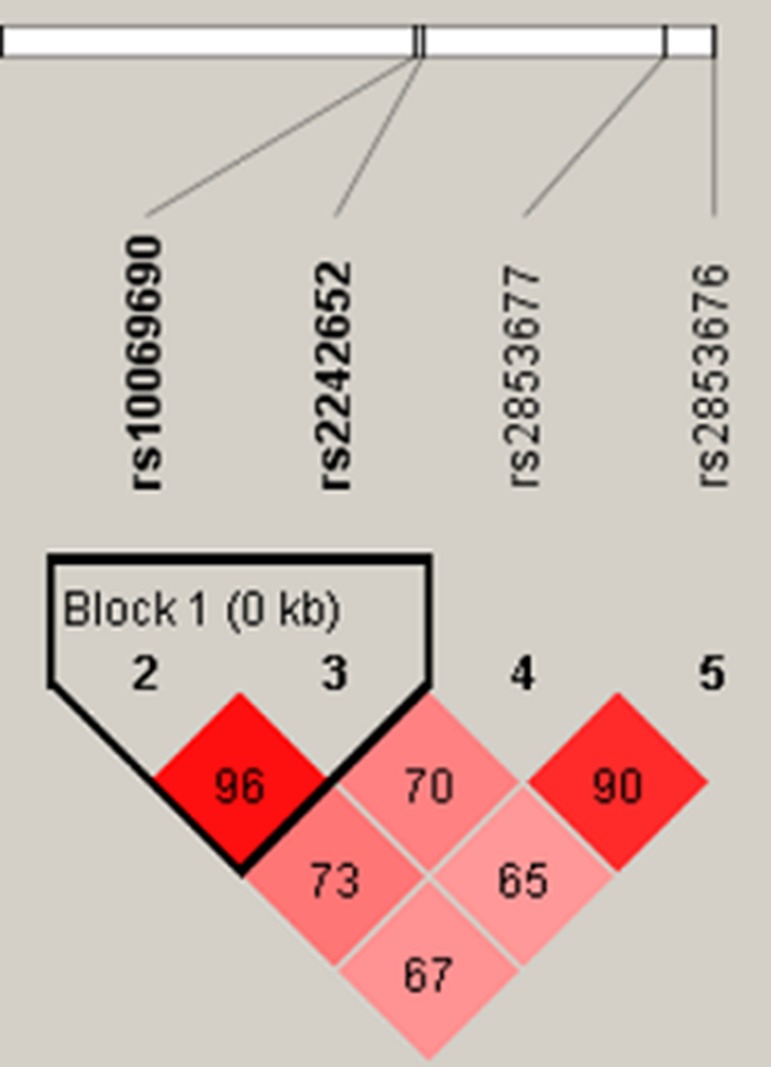
Haplotype block map for the four SNPs in *TERT* The LD between each pair SNPs is standardized D′, bright red corresponding to s very strong LD; white corresponding to no LD; pink corresponding to intermediate LD.

The two SNPs rs10069690 and rs2242652 in the *TERT* gene showed significant linkage disequilibrium, as shown in Figure 1. In order to assess the associations between SNP haplotypes and CHB risk, a Wald test was performed using an unconditional multivariate regression analysis. The frequency of haplotype “TA” in the case group was significantly higher than that in the health control group. The haplotype “TA” was found to be associated with an increased risk of CHB (adjusted OR = 1.58, 95% CI: 1.05–2.38, *P* = 0.027) adjusted by gender, age, smoking and drinking, as shown in Table 4.

**Table 4:** Haplotype frequencies and associated with CHB risk

SNPs	Haplotype	F_A	F_U	OR (95% CI)	*P*
rs10069690|rs2242652	TA	0.1777	0.1385	1.58 (1.05–2.38)	0.027
	CG	0.8161	0.8395	0.77 (0.52–1.14)	0.194

F_A: Frequency in case, F_U: Frequency in control, OR: Odds ratio, 95% CI: 95% Confidence interval *P*-values were adjusted by gender, age, smoking and drinking. *P* < 0.05 indicates statistical significance.

## DISCUSSION

In this case-control study, we investigated the association between SNPs in the *TERT* gene and the risk of CHB in a Chinese Han population. The results showed that rs10069690 was significantly associated with an increased risk of CHB in the dominant model and additive model. In addition, we found that the haplotype “TA” (rs10069690 and rs2242652) was associated with an increased risk of CHB.

TERT gene encodes the catalytic subunit of telomerase reverse transcriptase, which is really important component of telomerase. Several studies have reported that TERT regulates cell proliferation and metastasis, and that it also has a strong effect on alternative splicing and genetic control of telomere length [21, 22]. The variants of the TERT has been found to be associated with many types of cancers and aging-related disorders, including glioma [17], lung cancer [19], breast cancer [12], gastric cancer [23], astrocytoma [18] and so on. It has been demonstrated that the genotype TT of *TERT* polymorphism rs2736098 was associated with a decreased risk of CHB in Chinese males [14]. In this study, we found that *TERT* gene polymorphism rs10069690 and haplotype “TA” were associated with CHD risk in a Chinese Han population. We suggested that TERT gene may have association with cancers and disease by influencing the balancing the telomere length. In addition, Telomerase or TERT and telomeres have long been recognized to play pivotal parts in regulating immunological activity [24]. It is thus likely that *TERT* variants modify risk of CHB by influencing host immune function. However, the mechanistic details have not yet been elucidated.

Recently, it was reported that the T allele of rs10069690 was associated with an increased risk of breast cancer [12], lung cancer [19], esophageal cancer [23], gastric cancer [25], coronary heart disease [26]. However, rs10069690 was found to be associated with significantly reduced risk of hepatocellular carcinoma, prostate cancer [27]. We didn’t observe any association between the three SNPs (rs2242652, rs2853677and rs2853676) and CHD risk. Previous studies found that the three SNPs were also associated with several cancers risk [17, 23, 25, 28]. To date, there has been little report on the correlation between the polymorphisms of *TERT* and CHB risk. To our knowledge, this is the first report demonstrating an association between the variant rs10069690 and CHB risk, which need to be confirmed in further studies.

It should be noted that this study has limitations. First, the sample size is not large enough, and the CHB patients and controls included only Chinese population lived in Shaanxi Province. Second, the functional assessment of *TERT* genetic variants was not performed. Finally, the potential influences of environmental factors on the results could not be completely eliminated. Therefore, future prospective studies are required to confirm these findings, and the interaction of genetic and environmental factors in the development of CHB, and determine the functional role of these SNPs, with a larger sample.

In conclusion, our study identified that rs10069690 and haplotype “TA” in *TERT* gene are associated with a significantly increased risk of CHB, which may have the potentially to serve as prognostic biomarker for CHB in the Han Chinese population. Further validation of the functionality of the variants and its association with risk of CHB in other ethnic populations is warranted.

## MATERIALS AND METHODS

### Participants

A total of 542 unrelated Chinese Han individuals consisting of 242 CHB patients and 300 healthy controls were recruited in the case-control study. All the patients were recruited from the Haikou people’s Hospital, and diagnosed as a clinical disease of CHB according to the diagnostic criteria of CHB based on history of hepatitis B virus (HBV) infection, HBsAg/anti-HBs, HBeAg/anti-HBe and anti-HBc serostatus, HBV DNA level, biochemical liver function, ultrasonography and/or computerized tomography (CT)/ magnetic resonance imaging (MRI) [29]. The patients with the family history of liver, renal, endocrine and cardiovascular disorders disease were excluded. The control subjects were randomly selected from physical examination center of the Haikou people’s Hospital during the same period, with negativity for HBsAg, HBcAg, anti-HCV and anti-HIV and with no abnormalities based on physical examination. All the subjects were Chinese Han individuals and their ancestors had lived in the region at least the three generations. A uniform questionnaire was used at enrollment including gender, age, smoking and drinking. The characteristics of the participants are shown in Table 1.

### Ethics statement

The collected blood samples and the protocol in this study were strictly conformed to the Declaration of Helsinki and were approved by the institutional ethical committee of the Haikou people’s Hospital. Written consents were obtained from all participants before participation in the study.

### Genotyping

Five milliliters of peripheral venous blood was obtained from each subject and collected in an EDTA tube, and then the blood samples were stored in a refrigerator at −20°C for DNA extraction. Genomic DNA was extracted from whole blood using the GoldMag-Mini Whole Blood Genomic DNA Purification Kit (GoldMag. Co. Ltd., Xi’an, China) based on the manufacturers’ instructions. DNA concentration and purity were evaluated using a spectrophotometer (NanoDrop 2000; Thermo Fisher Scientific, Waltham, MA, USA) at wavelengths of A260 and A280nm.

We selected four SNPs (rs10069690, rs2242652, rs2853677 and rs2853676) from previously reported *TERT* gene polymorphisms [17, 19, 23, 25, 28], and matched SNPs with MAF > 5% in the HapMap of the Chinese Han Beijing population selected for association analysis. The sequences of amplification and extension primers corresponding to each SNP were designed by the Sequenom MassARRAY Assay Design 3.0 Software (Sequenom, San Diego, CA, USA), as showed in Table 5. Genotyping of the SNPs were performed using the Sequenom MassARRAY platform (Sequenom, San Diego, CA, USA) according to the standard protocol recommended by the manufacturer. We used the Sequenom Typer 4.0 software to perform data management and analyses.

**Table 5:** The sequences of primers of each SNP

SNP-ID	2nd-PCRP	1st-PCRP	UEP
rs10069690	ACGTTGGATGATGTGTGTTGCACACGGGAT	ACGTTGGATGCCTGTGGCTGCGGTGGCTG	GGGATCCTCATGCCA
rs2242652	ACGTTGGATGAGGCTCTGAGGACCACAAGA	ACGTTGGATGACAGCAGGACACGGATCCAG	gtcgGAGGACCACAAGAAGCAGC
rs2853677	ACGTTGGATGGCAAGTGGAGAATCAGAGTG	ACGTTGGATGATCCAGTCTGACAGTCGTTG	gggtAATCAGAGTGCACCAG
rs2853676	ACGTTGGATGCAAAACTAAGACCCAAGAGG	ACGTTGGATGTGTCTCCTGCTCTGAGACC	agatGGAAGTCTGACGAAGGC

SNP: Single nucleotide polymorphism; PCRP: Polymerase chain reaction primer; UEP: unique base extension primer Sequences are written in the 5 ′ →3 ′ (left to right) orientation.

### Statistical analysis

All statistical analyses were performed using the SPSS 19.0 statistical software (SPSS, Chicago, IL) and Microsoft Excel. We used the Pearson’s χ^2^ test and Welch’s *t* test to evaluate the differences in the demographic characteristics between the cases and controls. The Hardy-Weinberg equilibrium (HWE) of each SNP was assessed in order to compare the expected frequencies of the genotypes in the controls using χ^2^ test. The allele frequencies of the two groups were compared with chi-square test, and to evaluate associations between the SNPs and risk of CHB in the four models (genotype, dominant, recessive, and additive). Linkage disequilibrium analysis and SNP haplotypes were analyzed using the Haploview software package (version 4.2). The relative risk was estimated by odd ratios (ORs) and 95% confidence intervals (CIs) using unconditional logistic regression analysis. The *P*-value < 0.05 was considered statistically significant and all statistical tests were two-sided.
